# MBD3 Localizes at Promoters, Gene Bodies and Enhancers of Active Genes

**DOI:** 10.1371/journal.pgen.1004028

**Published:** 2013-12-26

**Authors:** Takashi Shimbo, Ying Du, Sara A. Grimm, Archana Dhasarathy, Deepak Mav, Ruchir R. Shah, Huidong Shi, Paul A. Wade

**Affiliations:** 1 Laboratory of Molecular Carcinogenesis, National Institute of Environmental Health Sciences, Research Triangle Park, North Carolina, United States of America; 2 Integrative Bioinformatics, National Institute of Environmental Health Sciences, Research Triangle Park, North Carolina, United States of America; 3 SRA International, Inc., Durham, North Carolina, United States of America; 4 Department of Biochemistry and Molecular Biology, Georgia Health Sciences University, Augusta, Georgia, United States of America; Medical Research Council, Human Genetics Unit, United Kingdom

## Abstract

The Mi-2/nucleosome remodeling and histone deacetylase (NuRD) complex is a multiprotein machine proposed to regulate chromatin structure by nucleosome remodeling and histone deacetylation activities. Recent reports describing localization of NuRD provide new insights that question previous models on NuRD action, but are not in complete agreement. Here, we provide location analysis of endogenous MBD3, a component of NuRD complex, in two human breast cancer cell lines (MCF-7 and MDA-MB-231) using two independent genomic techniques: DNA adenine methyltransferase identification (DamID) and ChIP-seq. We observed concordance of the resulting genomic localization, suggesting that these studies are converging on a robust map for NuRD in the cancer cell genome. MBD3 preferentially associated with CpG rich promoters marked by H3K4me3 and showed cell-type specific localization across gene bodies, peaking around the transcription start site. A subset of sites bound by MBD3 was enriched in H3K27ac and was in physical proximity to promoters in three-dimensional space, suggesting function as enhancers. MBD3 enrichment was also noted at promoters modified by H3K27me3. Functional analysis of chromatin indicated that MBD3 regulates nucleosome occupancy near promoters and in gene bodies. These data suggest that MBD3, and by extension the NuRD complex, may have multiple roles in fine tuning expression for both active and silent genes, representing an important step in defining regulatory mechanisms by which NuRD complex controls chromatin structure and modification status.

## Author Summary

Chromatin structure is tightly regulated by multiple mechanisms; its dysregulation is associated with developmental abnormalities and disease. The Mi-2/nucleosome remodeling and histone deacetylase (NuRD) complex is proposed to regulate chromatin structure by changing the location and/or the chemical properties of the fundamental building block of chromatin, the nucleosome. NuRD has been shown by genetics to be important for normal development, yet the detailed mechanism of how NuRD regulates chromatin structure is still unclear. Here, we study the localization and function of MBD3, a component of NuRD, in two human breast cancer cell lines using two independent genomic technologies. Our data demonstrate that existing models, which associate NuRD with transcriptional repression, are not completely correct. Rather, MBD3 showed cell-type specific localization at active genes. Moreover, we found a previously unidentified localization of MBD3 across gene bodies and identified a regulatory role for MBD3 in nucleosome organization. Our data provide a reliable starting point from which to address mechanisms by which NuRD controls chromatin structure and nuclear biology.

## Introduction

Since its discovery in the late 1990's by a number of investigators, the Mi-2/nucleosome remodeling and histone deacetylase (NuRD) complex has been proposed to regulate chromatin structure and promote transcriptional repression via its intrinsic nucleosome remodeling and histone deacetylation activities [1]. Although this model provided a useful experimental framework, like all models, it has been challenged by subsequent data. In particular, the depiction of NuRD's principal function as a regulator of a silent chromatin state has been questioned by genetic and molecular studies from Georgopoulos and colleagues [2], [3], [4] and Hendrich and colleagues [5], which provide compelling evidence that NuRD can have both positive and negative impacts on gene expression.

zNuRD complex contains six core subunits which are invariably encoded by 2 or more gene paralogs, prompting the hypothesis that combinatorial assembly of subunits may contribute to functional specificity [6]. The smallest complex subunit can be either MBD2 or MBD3 [7], members of the family of proteins that possess the methyl CpG binding domain (MBD) fold. While MBD2 is a *bona fide* methyl CpG binding protein, mammalian MBD3 has lost the ability to selectively interact with methylated DNA [8]. Recently, however, it has been suggested that MBD3 specifically binds another modified form of DNA, 5-hydroxymethylcytosine (5-hmC), leading to NuRD recruitment at 5-hmC marked loci in embryonic stem cells [9]. Other investigators find that NuRD complexes are involved in different aspects of the transcription cycle, coupling its action to enhancers [10] or to recruitment of polycomb complexes [11]. Currently, the field lacks consensus on the localization of MBD3 (and by extension NuRD complex) in the genome as well as its roles in modulating chromatin biology to facilitate gene regulation [12].

Chromatin immunoprecipitation coupled to massively parallel sequencing, ChIP-seq, represents ‘gold standard’ methodology to identify sites of enrichment of a particular protein (or modified protein) across the genome. High quality ChIP-seq data are dependent on a number of technical factors, including antibody quality, fixation conditions (where ChIP is performed from fixed chromatin), chromatin shearing or cleavage with nuclease, and stringency of wash conditions for immune complexes. Progress over the last decade has established the principle that conditions that produce high quality ChIP data for one protein may not necessarily be effective for others. Performing ChIP on chromatin regulators, including NuRD complex, is particularly challenging [13]. Given the technical issues, the lack of concordance of recent NuRD ChIP-seq studies may not be surprising. It does, however, highlight a need for independent studies to provide additional data for comparison.

Here we have analyzed human MBD3 localization across the genome using two complementary genomic approaches. We first used DNA adenine methyltransferase identification (DamID), a methodology independent of antibodies, chromatin shearing and other technically challenging features of ChIP. We also performed ChIP-seq studies of endogenous human MBD3 following optimization of antibody, cross-linking and chromatin shearing. The results of these two techniques were in excellent agreement, suggesting they may not be unduly influenced by technical issues. The resulting, composite location analysis revealed unexpected association of MBD3 with the active fraction of the genome. MBD3 localized preferentially to active promoters characterized by histone marks associated with open chromatin and with genomic regions bearing the properties of enhancers, supporting the emerging evidence that models depicting NuRD's principal functions as a component of repressive chromatin require re-evaluation. In addition, MBD3 coated gene bodies of actively transcribed genes, extending to the transcript end site. Conversely, we also observed association of MBD3 with promoters of genes marked by repressive histone marks, albeit not with the frequency of active promoters, highlighting the likelihood that NuRD action is not unidirectional and is context dependent. Finally, the largest category of loci bound by MBD3 has no obvious association with known chromatin features, underscoring the relative dearth of knowledge on NuRD localization and function.

## Results

### MBD3 localization by DamID

**Figure 1:**
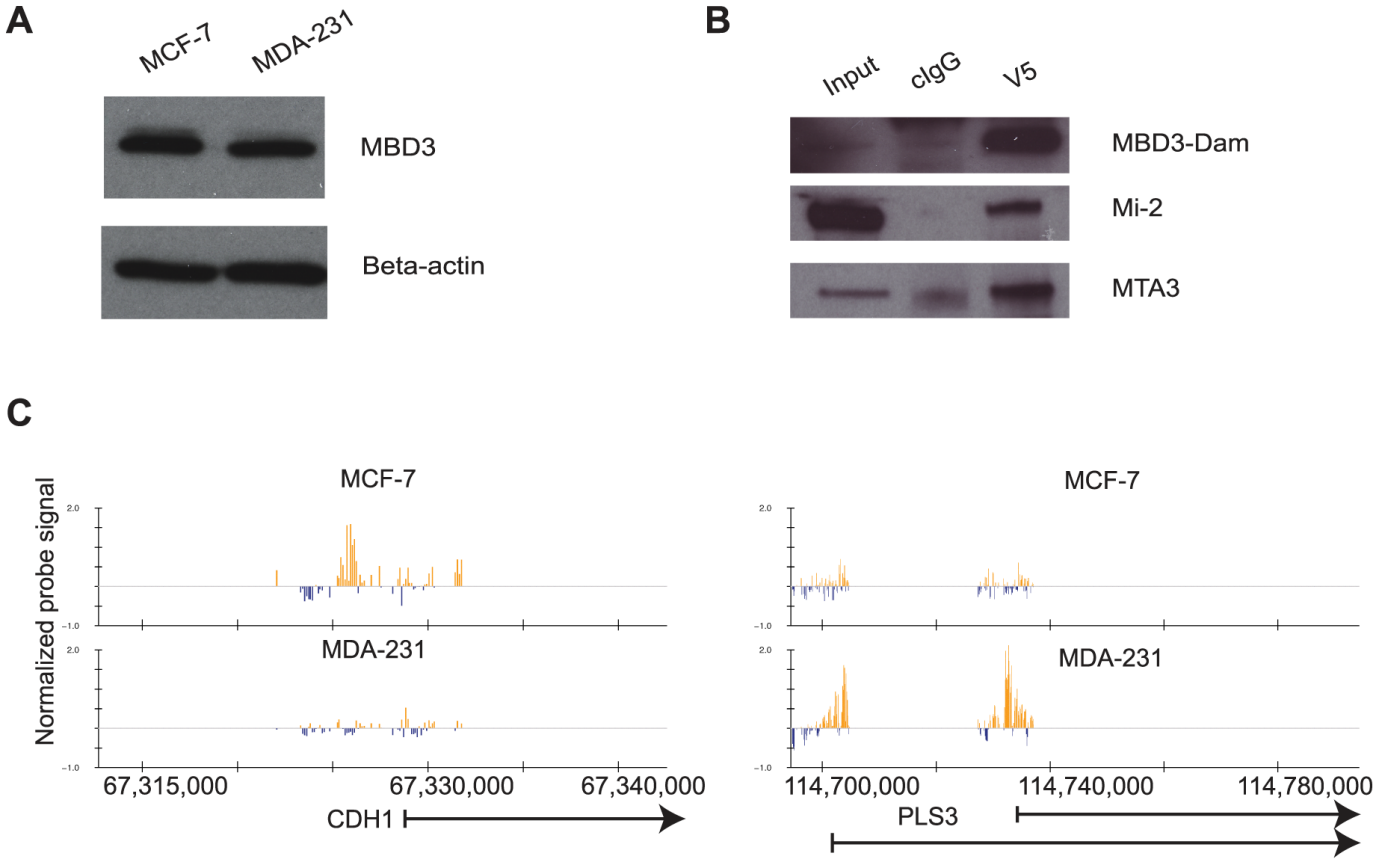
The DamID platform for location analysis of human MBD3. A. MCF-7 and MDA-231 cells express endogenous MBD3 at equivalent levels. The immunoblot depicts endogenous MBD3 protein expression in MCF-7 and in MDA-231 cells. Beta actin is included as a control. B. Dam-fused MBD3 can be incorporated into the NuRD complex. An IP-western analysis was performed on lysates from HeLa cells infected with the pLgw-MBD3-V5-EcoDam using V5 antibody or control IgG. The resulting immunoprecipitates were compared to input lysates and probed for MBD3-Dam fusion and endogenous Mi-2 and MTA3. C. Genome browser (hg18, http://genome.ucsc.edu, [53]) views of exemplar genes analyzed by DamID. The location of genes is indicated at the bottom of the respective panels. Normalized probe signal within the regions displayed is indicated above the genomic coordinates (hg18).

We initiated location analysis of MBD3 in human cells using the DamID technique [14]. To facilitate biological comparisons, we chose two human breast cancer cell lines with different biological properties. MCF-7 cells provide a model for the luminal class of breast tumors, MDA-MB-231 (MDA-231) cells model basal tumors [15]. As a prelude to location analysis, we first confirmed that the cell lines chosen express comparable levels of endogenous MBD3, and that the exogenously expressed MBD3-Dam fusion protein was expressed, nuclear, and could be incorporated into NuRD complex (Figure 1, Figure S1).

**Figure 2:**
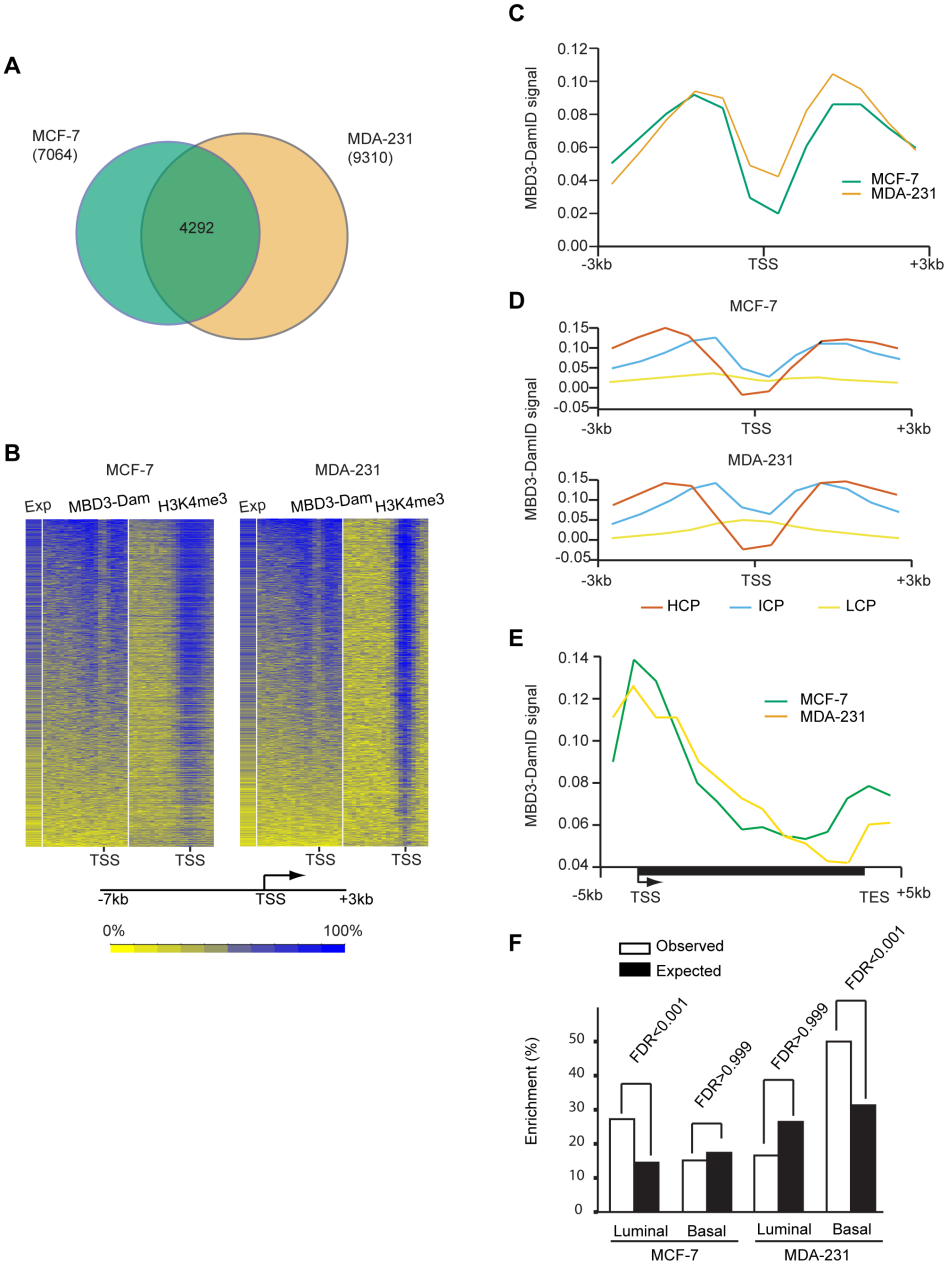
MBD3 localizes to active CpG rich promoters independent of cell type. A. The Venn diagram depicts the overlap of transcripts with an MBD3 peak within 3-7 and 9,310 in MDA-231 were included in the overlap analysis. A total of 4292 transcripts had MBD3 peaks at TSS in both cell lines. B. The heatmap displays MBD3-DamID signal, H3K4me3 signal (MCF-7 ChIP-seq data is from UW, GEO accession number GSM945269, ENCODE Project Consortium 2011; MDA-231 ChIP-seq, this study) and gene expression in MCF-7 and MDA-231 [54]. 8,207 genes were selected for display as described in Methods. Genomic intervals from −7 to +3 kb relative to TSS were binned into 20 equal bins and ranked by MBD3-DamID signal. H3K4me3 and gene expression were displayed in the same order. The color scale for interpretation of signal intensity is located at the bottom of the heatmap. C. Refseq transcript 5′ ends were selected as described in Methods. The plot depicts average DamID signal for all transcripts across the interval from −3 to +3 kb relative to TSS. MCF-7 and MDA-231 plots are in green and tan, respectively. D. For each cell type, promoters were subdivided as in [16]. The plot displays the average DamID signal for each promoter class by cell type across the indicated genomic interval. Promoter class is indicated by color coding as indicated in the figure. E. A composite gene model for genes selected as described in Methods. DamID signal intensity was averaged for genes in the two cell lines and displayed in the plot. The location of TSS and TES is indicated. Cell lines are distinguished by color coding as indicated. F. The column graph depicts the enrichment percentage derived from Functional Analysis. Genes were selected as MBD3 targets if there was an MBD3 peak within 3 kb of TSS. The luminal and basal discriminatory genes were as described [47].

We prepared two biological replicates of Dam alone and MBD3-Dam fusion for each cell line and performed 2 color hybridization using human promoter arrays. The raw data were processed as described in Methods and the ratio of MBD3-Dam to Dam alone displayed in genome browser format. Visual inspection of the data indicated multiple areas where the two cell types displayed highly similar patterns of localization as well as regions where localization differed by cell type (Figure 1C). We determined local enrichment in the two cell lines by defining peaks using a conventional peak-calling algorithm (see Methods). Peak localization was consistent across biological replicates (greater than 75% overlap) and differed across cell type (less than 50% overlap) suggesting the data were of high quality. We identified putative NuRD target genes as those containing a peak within 3 kb of the annotated transcription start site, resulting in 7,064 putative targets in MCF-7 cells, 9,310 in MDA-231 cells. Comparison of target genes across cell type indicated many common targets and many cell-type specific targets (Figure 2A). Collectively, these data indicate that MBD3, and by extension NuRD complex, has a cell-type specific localization pattern, suggesting cell-type specific functions.

To assess the relationship of MBD3 localization to gene expression and histone modification patterns, we merged the biological replicates for each cell type, binned promoters into 20 bins from −7 to +3 kb relative to transcription start site (TSS), and calculated occupancy for MBD3 and for H3K4me3 (assessed by ChIP-seq) as described in Methods. We displayed the results in a heatmap with genes ordered based on MBD3 density; scores for H3K4me3 and for gene expression were displayed in the same order. We observed a striking association of MBD3 DamID score with H3K4me3 density by ChIP-seq and with gene expression – regardless of cell type (Figure 2B). Most MBD3 bound genes were highly expressed and carried high levels of H3K4me3 in both MCF-7 and MDA-231 cells, suggesting MBD3 preferentially associates with open chromatin regions at actively transcribed genes.

To describe the binding pattern of MBD3-Dam with promoters, we constructed a composite analysis across all promoters (−3 to +3 kb relative to TSS). MBD3 was prominently enriched in two distinct peaks, each about 1.5 kb from TSS, with a prominent dip at TSS in both cell lines (Figure 2C). To ascertain whether this enrichment pattern was associated with promoter type, we subdivided our MBD3 data into three promoter classes [16]. Regardless of cell line, we observed that MBD3 preferentially associates with CpG rich promoters with little obvious accumulation evident at CpG poor promoters (Figure 2D).

To develop an understanding of the association of MBD3 with genic regions not represented on the promoter arrays, we performed MBD3 DamID using a tiling array covering human chromosomes 6, 7, and 8. We constructed a gene model (see Methods), observing a gradient of MBD3 density from a peak around TSS that gradually declines across the gene body. A second peak of MBD3 accumulation was noted roughly concurrent with the transcript end site (TES) (Figure 2E).

The association of MBD3 with overlapping, but distinct, genes in MCF-7 and MDA-231 cells suggested that the MBD3-NuRD complex may play a role in cell-type specific patterns of transcription. To address this issue, we performed Functional Analysis using Broad Institute's Molecular Signature Data Base (MSigDB v 3.0). We specifically asked whether the MBD3-NuRD target genes (as defined for Figure 2A) were enriched for gene expression patterns diagnostic of the luminal and basal transcriptional programs in breast cancer (Figure 2F, Table S1). Functional analysis demonstrated significant enrichment of luminal discriminatory genes, but not basal discriminatory genes, in the MCF-7 (luminal) specific MBD3-bound gene set. MDA-231 (basal) specific MBD3-NuRD putative targets displayed the opposite pattern. These data suggest that MBD3, and by extension the MBD3-NuRD complex, may have a biological role in breast cancer subtype specification.

### High-resolution mapping of MBD3 by ChIP-seq

The DamID technique, while extremely useful, has relatively low resolution, relies on the presence of the GATC motif, and our experiments provided data only on the regions tiled in the microarray platform chosen. Therefore, we opted to pursue MBD3 ChIP-seq to obtain a higher resolution map for MBD3 that included genic and intergenic regions not represented on the arrays. We first optimized conditions for ChIP of endogenous MBD3, finding that fixation conditions are critical to the success of robust MBD3 ChIP. We applied a two-step crosslinking method, similar to our previous conditions [17], [18], crosslinking with disuccinimidyl glutarate followed by formaldehyde (see Methods).

**Figure 3:**
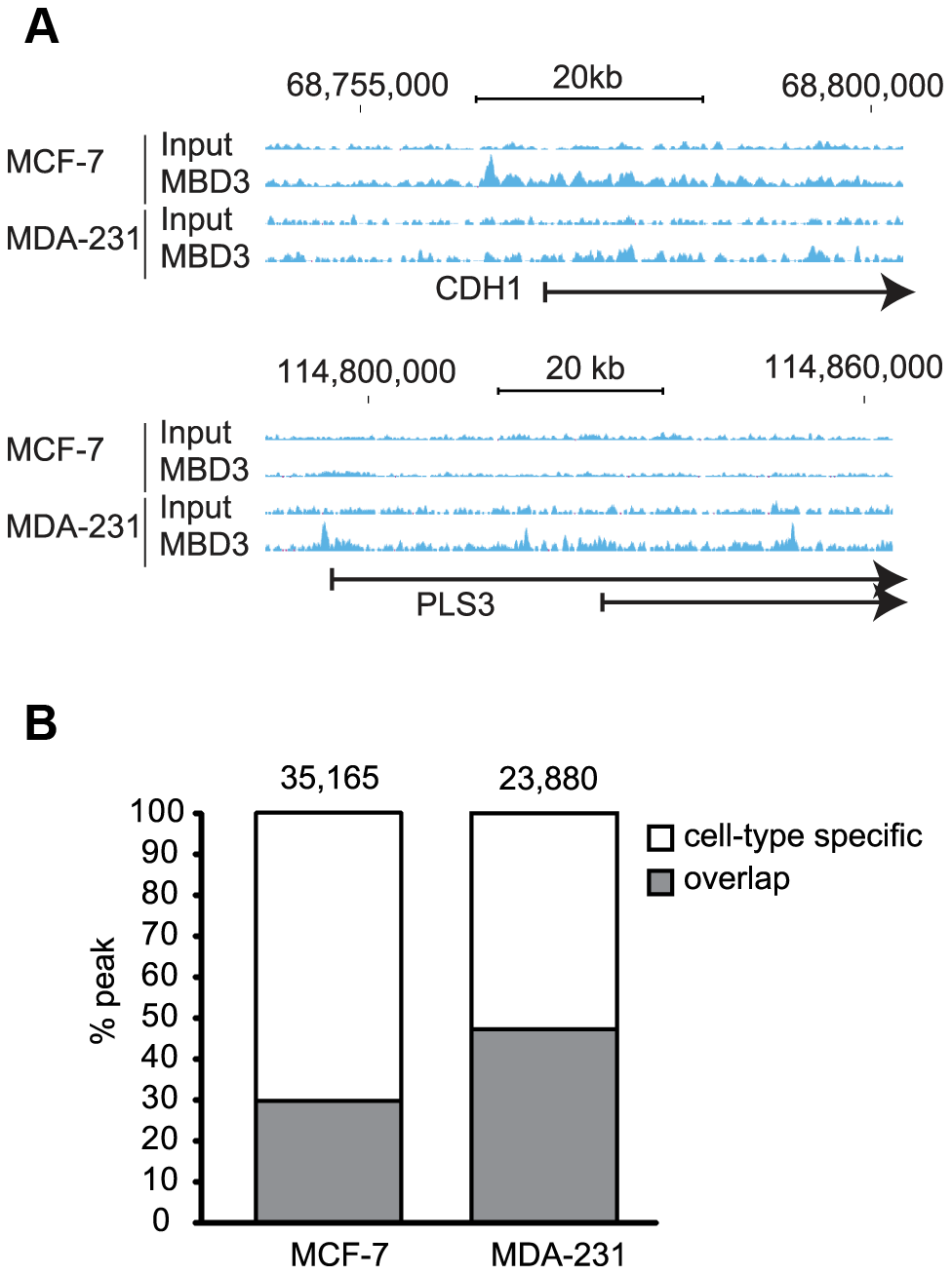
MBD3 localizes in a cell-type specific manner by ChIP-seq. A. Exemplar loci are depicted in genome browser format (hg19, http://genome.ucsc.edu, [53]). Individual sequencing tracks are indicated to the left of the browser view. Genomic intervals and scale bar are indicated above the tracks. B. The column graph depicts the total number of peaks defined from the current study in each cell type. The colors depict peaks that overlap in the two cell types by at least one base as well as those with no overlap (cell-type specific in the figure).

Using these optimized conditions, we prepared two biological replicates of MBD3 ChIP in MCF-7 and in MDA-231 cells. Precipitated DNA was analyzed by massively parallel sequencing. After initial filtering and mapping of the sequence data, we merged the biological replicates for each cell line prior to further analysis (see Methods). Visual assessment in genome browser format indicated multiple regions where enrichment appeared similar in both cell lines as well as many regions where enrichment was cell-type specific (Figure 3A). We assessed local enrichment of MBD3 more rigorously by defining peaks using SICER [19]. We detected 35,165 and 23,880 peaks in MCF-7 and MDA-231 cells, respectively; peak overlap between the two cell lines was similar to the pattern observed in the DamID analysis (Figure 3B). Finally, we formally compared peaks detected in ChIP-seq to DamID and observed excellent concordance (Table S2, Figure S2).

**Figure 4:**
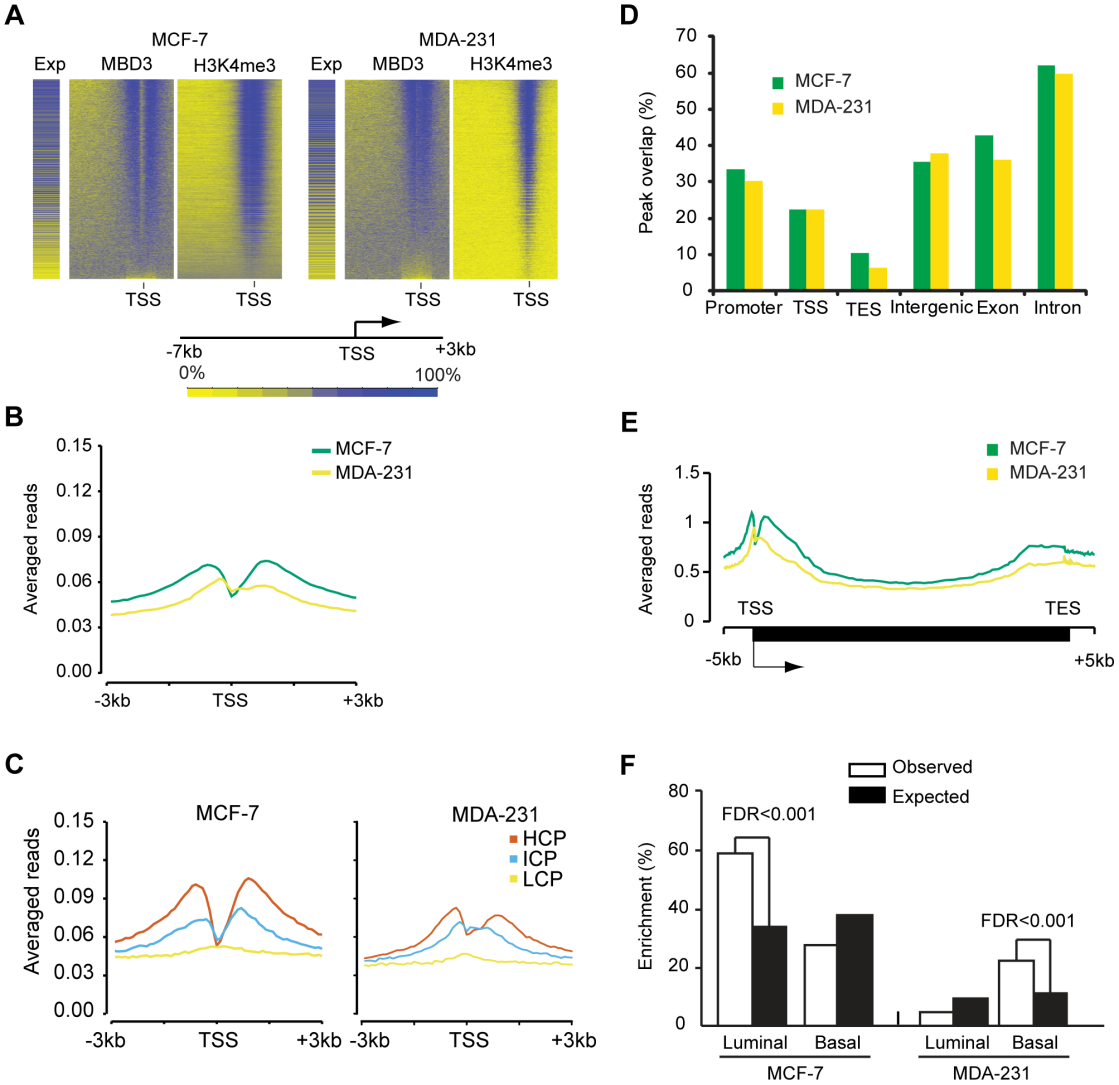
MBD3 localizes to active promoters and to other genomic regions. A. The heatmap was prepared in a manner similar to Figure 2B. Genes were binned (500 bp per bin) from −7 to +3 kb relative to TSS, and rank ordered by MBD3 density. H3K4me3 and gene expression were displayed in the same order. Intensity signal scale is indicated in the figure. B. Refseq transcript 5′ ends were selected as described in Methods. The plot depicts average MBD3 ChIP-seq signal for all transcripts across the interval from −3 to +3 kb relative to TSS. Color coding is as depicted in the figure. C. For each cell type, promoters were subdivided as in [16]. The plot displays the average MBD3 ChIP-seq signal for each promoter class by cell type across the indicated genomic interval. Promoter class is indicated by color coding as indicated in the figure. D. The column graph depicts the overlap of MBD3 peaks with the indicated genomic features. Peaks that overlap promoters (−3 to +3 kb relative to TSS) or the indicated features by at least one base are included in the columns. Note that a single peak could overlap with more than one type of feature in this analysis. E. A composite gene model for genes selected as described in Methods. MBD3 ChIP-seq signal intensity was averaged for genes in the two cell lines and displayed in the plot. The location of TSS and TES is indicated. Cell lines are distinguished by color coding as indicated. F. The column graph depicts the enrichment score derived from Functional Analysis. Genes were selected as MBD3 targets if there was an MBD3 peak within 3 kb of TSS. The luminal and basal discriminatory genes were as described [47].

Like the DamID data, higher MBD3 density by ChIP was observed at a large number of promoter regions. As with DamID, we visualized the MBD3 localization data by binning promoters (500 bp bins, −7 to +3 kb relative to TSS) and ordering genes by MBD3 density. We once again observed a strong association of MBD3 with actively transcribed genes marked by H3K4me3 (Figure 4A). As was the case with DamID, metagene analysis indicated prominent peaks of MBD3 localization flanking a prominent dip in density at the TSS with a preferential association with CpG rich promoters, although the resolution of ChIP-seq was clearly superior (Figure 4B, 4C).

The ChIP-seq data permitted us to analyze regions not covered in depth by the DamID analysis. We assessed the overlap of MBD3 peaks with genomic features, finding that MBD3 overlapped with promoters (−3 to +3 kb relative to TSS) and with TSS with high frequency (Figure 4D). As was the case with DamID, we also observed frequent peak overlap with TES's. MBD3 tended to be more frequently associated with genes (exons and introns) than with intergenic regions, although localization between annotated genes is a common event (Figure 4D). Metagene analysis (Figure 4E) indicated that MBD3 peaks were found in abundance at or near the TSS with a 5′ to 3′ gradient across the gene body and a second, less pronounced, peak concurrent with the TES. As was the case with DamID data, genes marked by MBD3 in MCF-7 cells were enriched in transcripts defining the luminal gene expression pattern in breast cancer; genes marked by MBD3 in MDA-231 cells were enriched in transcripts defining the basal transcriptional program (Figure 4F, Table S3). Collectively, the ChIP-seq data indicate that MBD3 is predominantly found at actively transcribed genes with CpG island promoters and that the protein coats the gene body, extending to the TES.

### Local chromatin features of MBD3 bound regions

**Figure 5:**
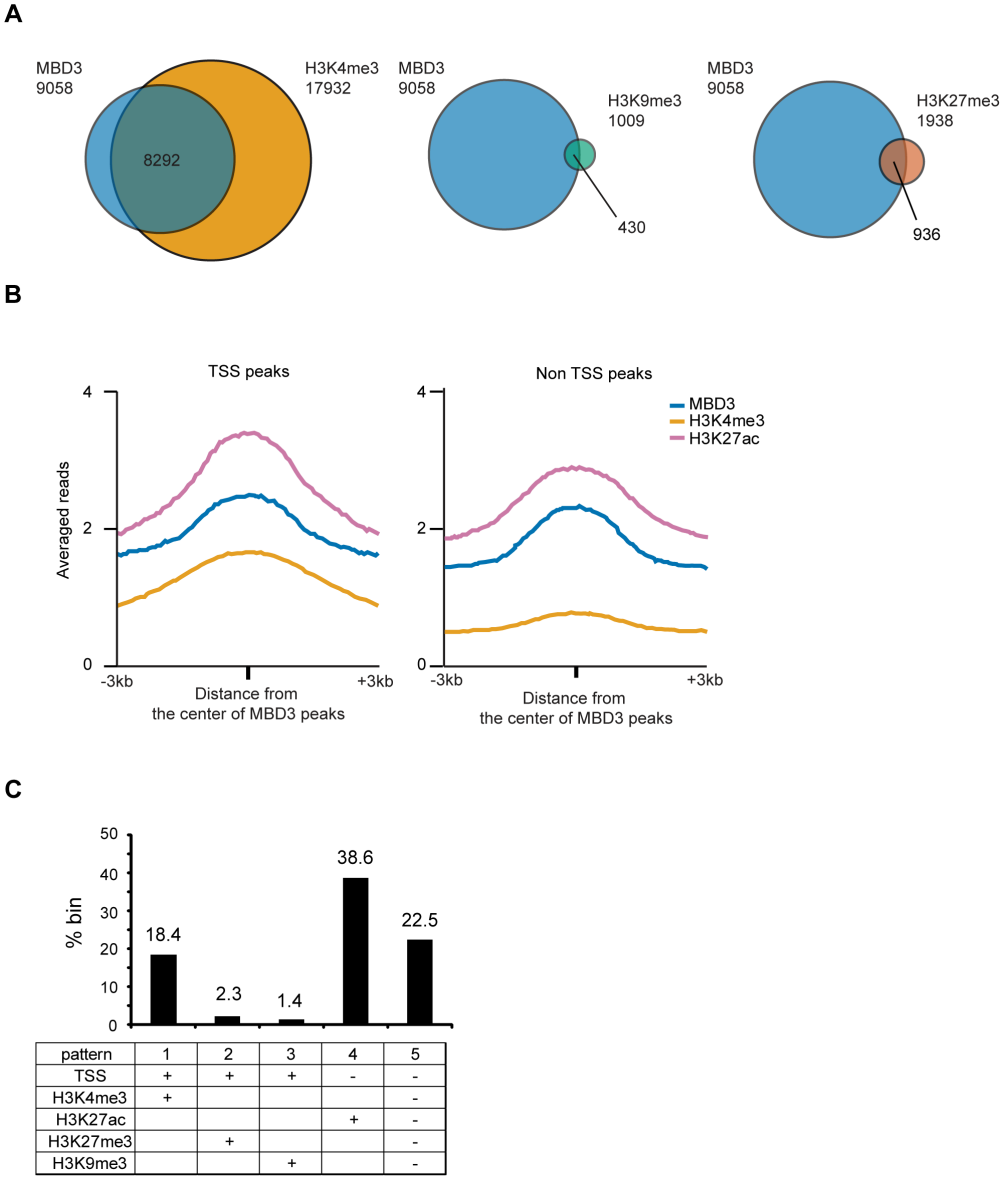
Association of MBD3 in MCF-7 with local histone modifications. A. The Venn diagrams depict overlap of MBD3 associated TSS's with those marked by H3K4me3, H3K9me3, or H3K27me3 (as described in Methods). The total number of TSS's in each category is indicated in the figure. B. The line graphs depict abundance of MBD3, H3K4me3 and H3K27ac (ChIP-seq data from USC/Farnahm, GEO accession number GSM945854, ENCODE Project Consortium 2011) at MBD3 peaks overlapping TSS or MBD3 peaks not overlapping TSS. C. The column graph depicts the percentage of 1 kb genomic bins bound by MBD3 that display the indicated modification/location patterns. (Presence of MBD3 or histone marks is assigned as described in Methods).

We utilized publicly available data from the ENCODE project [20] collected in MCF-7 cells to ascertain the nature of chromatin bound by MBD3. Focusing first on transcript 5′ ends, we noted that transcripts in which an MBD3 peak overlaps the promoter region (−3 to +3 kb relative to TSS) were associated with a peak of H3K4me3 91.5% of the time (Figure 5A). MBD3 bound promoters were associated with H3K9me3 very infrequently, approximately 4.7% of the time (although this does represent a substantial portion of H3K9me3 bound promoters −42.6%). A significant number of MBD3 bound promoters were packaged in chromatin characterized by the presence of H3K27me3 (10.3% of MBD3 associated promoters, 48.3% of H3K27me3 associated promoters) in agreement with data from Hendrich and colleagues in murine ES cells [11].

Given the prevalence of MBD3 peaks in intergenic regions, we asked whether these loci bore chromatin signatures indicative of function. We plotted the distributions of MBD3 and histone modifications (H3K4me3 and H3K27ac) relative to the MBD3 peak center. Non-TSS MBD3 colocalized with H3K27ac but not with H3K4me3, while MBD3 colocalized with both H3K4me3 and H3K27ac around TSS (Figure 5B). Thus, non-TSS MBD3 bound regions, on average, bear the chromatin signature of active enhancers, a genomic feature that has been associated with NuRD complex in ES cells [10].

Next, we quantified the overlap of MBD3 with various patterns of histone marks across the genome by dividing the human genome into 1 kb windows and calculating the enrichment for several chromatin marks in each window (see Methods). Overall, we noted at least five different patterns of histone modification characteristic of MBD3 bound genomic regions (Figure 5C). Regions encompassing the transcription start and containing histones modified by H3K4me3 (pattern 1; active promoters) were very abundant in this dataset, accounting for 18.4% of MBD3 enriched regions. Colocalization with H3K27me3 and TSS (pattern 2; inactive promoters) was present with some frequency (2.3% of all MBD3 enriched regions), while colocalization with H3K9me3 (pattern 3; inactive promoters) was rare (about 1.4%) in these data. Regions enriched in both MBD3 and H3K27ac, but not overlapping TSS (pattern 4; active enhancers), were the most frequent pattern observed in our dataset (38.6%). Surprisingly, approximately one quarter (22.5%) of MBD3 enriched genomic regions (pattern 5) were found in chromatin lacking any of these patterns of histone marks, suggesting that much remains to be learned regarding NuRD complex enrichment relative to local chromatin features.

To clarify whether MBD3 localization has any correlation with DNA modification in this system, we queried DNA methylation status within MBD3 bound regions. In murine ES cells, Yildirim and colleagues suggested a causal relationship between 5-hmC modification and NuRD localization [9]. We assessed the methylation status of MBD3 bound CpG islands (using ENCODE reduced representation bisulfite sequencing data in MCF-7 cells). The majority of CpG islands overlapping a peak of MBD3 were hypomethylated with 60% of these islands falling within the lowest decile of DNA methylation (Table 1, Figure S3). MBD3 was enriched at islands falling in the lowest two deciles of DNA methylation and excluded from the highest 3 (Table 1). These data indicate that MBD3 binds preferentially to unmethylated CpG islands in human breast cancer cells and agrees with recent biochemical studies indicating that MBD3 has no measurable biochemical preference for methylated cytosine [21], [22].

**Table 1:** Methylation level of CpG island with an MBD3 peak that fall into within the indicated levels of DNA methylation as determined by RRBS.

Methylation levels (x)	MBD3 associated CpGs	Total CpGs	P value
x = 0%	538 (4.33%)	1023 (4.73%)	0.08758
**0<x<10%**	**7178 (57.71%)**	**10348 (47.89%)**	**<2.2e-16**
**10≤x<20%**	**522 (4.20%)**	**775 (3.59%)**	**0.005042**
20≤**x**<30%	324 (2.60%)	514 (2.38%)	0.207
30≤**x**<40%	259 (2.08%)	431 (1.99%)	0.6071
40≤**x**<50%	213 (1.71%)	358 (1.66%)	0.7319
50≤**x**<60%	219 (1.76%)	398 (1.84%)	0.6189
60≤**x**<70%	220 (1.77%)	464 (2.15%)	0.01847
**70≤x<80%**	**252 (2.03%)**	**639 (2.96%)**	**2.66E-07**
**80≤x<90%**	**584 (4.70%)**	**1507 (6.97%)**	**<2.2e-16**
**90≤x≤100%**	**2129 (17.12%)**	**5153 (23.85%)**	**<2.2e-16**
Total	12438 (100.00%)	21610 (100.00%)	

CpG islands bound by MBD3 (see Methods) were binned by methylation level (see Methods). Enrichment or depletion of MBD3 in each bin was determined by two-tailed t-test. Significantly enriched or depleted bins (p<0.001) are highlighted in bold.

### MBD3 peaks in intergenic regions are in physical proximity to promoters

**Figure 6:**
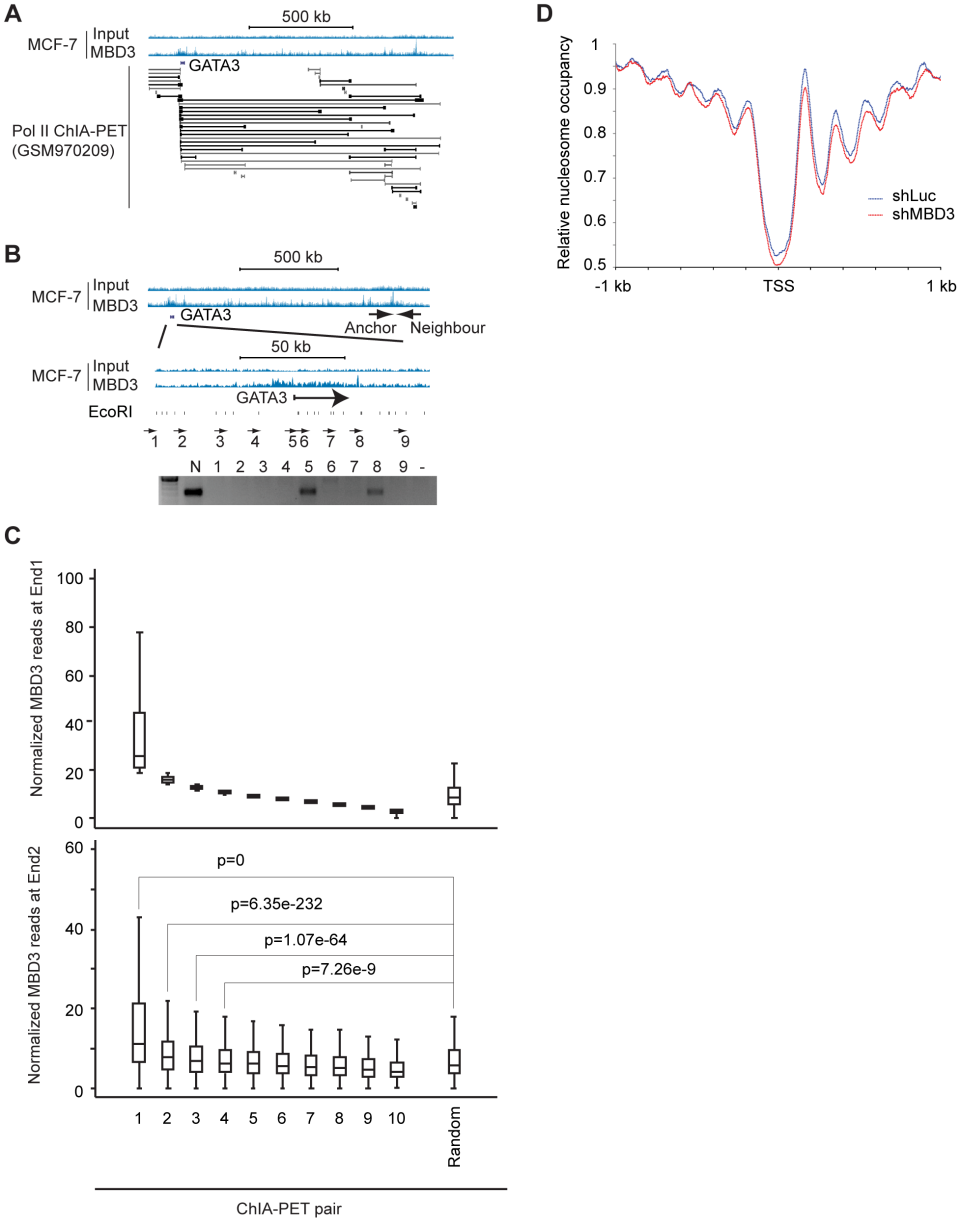
Non-TSS MBD3 peaks overlapping H3K27ac are in proximity to promoters. A. The genome browser view displays the genomic region around the human GATA3 locus. MBD3 ChIP-seq data is depicted at the upper portion of the panel along with a scale bar. ChIA-PET read pairs derived from GSM970209 are depicted in the lower portion of the panel. B. The genome browser view displays the same genomic region of the GATA3 locus depicted in panel A. The location of the anchor primer is depicted in the upper panel of tracks. The region indicated is expanded in the lower set of tracks to display primer sets around GATA3. The location of EcoRI sites within the region is indicated below the tracks. The gel displays PCR products using the anchor primer in PCR with the indicated primers tiling across GATA3. C. MBD3 read depth was plotted for all genes where Pol II ChIA-PET pairs exist with one end anchored at TSS (see Methods). ChIA-PET pairs were rank ordered by MBD3 density at the TSS end (end 1) and divided into 10 groups. The upper panel depicts MBD3 density for the end 1. The lower panel depicts MBD3 density at the distal end (end 2) of the ChIA-PET pair. D. Metagene plots (see Methods) of nucleosome occupancy from cells with and without depletion of MBD3.

Distal enhancer elements physically interact with promoter regions and these interactions have a major role in gene regulation [23]. A substantial portion of MBD3 bound loci have chromatin features consistent with function as enhancers. The spatial architecture of the nucleus and proximity of distal regulatory elements to promoters relative to occupancy of a given protein is conveniently measured by ChIA-PET [24]. Given the high frequency with which we observed MBD3 peaks at the TSS of active genes, we assessed the relationship of MBD3 occupancy to RNA polymerase II and to distal regulatory DNA by querying a Pol II ChIP-PET data set in MCF-7 cells. We observed frequent occurrence of MBD3 enrichment at both ends of Pol II ChIA-PET pairs. An exemplar locus, GATA3 is depicted in Figure 6A. We used 3C technology [25] to validate whether a selected subset of MBD3 bound intergenic peaks coinciding with Pol II ChIA-PET ends, including loci in the vicinity of GATA3, NR2F2, NRIP1, and MASTL in MCF-7 cells, are in proximity to their respective promoters in three-dimensional space. At all 4 loci queried, we detected an interaction between a distal MBD3 bound peak and the promoter region (Figure 6B, Figure S4).

To extend these observations to the level of the entire dataset, we plotted MBD3 density at TSS for genes with a Pol II ChIA-PET pair anchored at TSS, ranking genes by MBD3 abundance. Display of MBD3 abundance at the distal ChIA-PET pair in the same order revealed that genes with high level MBD3 at TSS tend to have high MBD3 at the distal region defined by Pol II ChIA-PET as being in proximity in three-dimensional space (Figure 6C). These data show that some intergenic MBD3/H3K27ac peaks are in physical proximity to core promoters in three dimensional space, consistent with action as enhancers.

### MBD3 affects nucleosome occupancy at promoter and enhancer regions

Because MBD3 is a component of NuRD complex which contains chromatin remodeling factors (CHD3 and CHD4), we hypothesized that MBD3 regulates nucleosome occupancy at its binding sites. To test this hypothesis, we mapped the nucleosome positions in MBD3 depleted and control MCF-7 cells. We verified that MBD3 was efficiently depleted (Figure S5). Native chromatin from control and MBD3 depleted cells were digested with micrococcal nuclease and the resulting mononucleosome-sized DNA fragments were collected and subjected to massively parallel sequencing. We identified about 150 million nucleosomes for each group and performed a metagene analysis centered on TSS (Figure 6D, Table S4). Control cells showed a regular nucleosome organization consistent with previous publications; a nucleosome depleted region (NDR) is observed around the TSS and well-positioned nucleosomes flank this NDR. In MBD3 depleted cells, nucleosome phasing was similar to that in control cells but nucleosome occupancy was decreased - particularly at the NDR, and the −1, +1, +2, +3, and +4 nucleosomes. To test whether this effect is MBD3 dependent, we ranked promoters based on MBD3 occupancy (Figure S6). The changes at the NDR and +1nucleosome did not correlate with MBD3 occupancy and may be indirect. However, the occupancy pattern at −1, +2, +3, and +4 nucleosomes differed substantially upon MBD3 depletion at promoters in the highest quartile while these same nucleosomes did not change upon MBD3 depletion in the lowest quartile. These data indicate that MBD3 regulates nucleosome organization, particularly near promoters and in gene bodies that have high MBD3 occupancy.

## Discussion

Chromatin regulators are critical integration points wherein biological signals are converted into alterations in gene expression. These protein machines are essential to normal cell function, to development and to differentiation [26]. A large number of chromatin regulators are mutated in cancer, highlighting the importance of their function in normal cells [27]. Critical to understanding the biology of these regulators is determination of their sites of accumulation, and presumably of their action, within the genome. Here, we have utilized two complementary techniques to address the localization of MBD3, and by extension NuRD complex, arriving at a robust and reliable location map. These data associate MBD3 with previously undescribed genomic features, including extensive colocalization with the bodies of active genes. Further, an abundant category of MBD3 localization observed was not associated with any particular genomic feature, histone or DNA mark we analyzed, suggesting that the catalog of functions for NuRD is not yet exhaustive. Finally, we assessed the contribution of MBD3 to chromatin organization, finding that MBD3 regulates nucleosome organization near promoters and within gene bodies - consistent with its localization.

The use of multiple techniques for location analysis of chromatin associated factors provides an opportunity to control for common technical problems inherent to a single protocol. DamID involves expression of a fusion protein that modifies DNA in its genomic vicinity with detection relying on creation of novel restriction sites. It suffers from poor spatial resolution relative to ChIP and the necessity of exogenous expression of a fusion protein that must faithfully recapitulate the biological properties of the unmodified factor. Chromatin immunoprecipitation relies on biochemical fractionation of chromatin, in many cases following fixation. High quality ChIP results are reliant on antibody affinity and specificity as well as on crosslinking conditions and biochemical fractionation methods. Our results using these two techniques are in excellent agreement, suggesting they are converging on a robust genomic location map for MBD3 in the system chosen. Given the poor concurrence and quality issues (Table S5) of recent NuRD ChIP-seq data [12], the convergence of location determined by independent techniques provides clarity to the question of where the enzyme is enriched in the genome.

Localization of MBD3 at promoter regions of active genes marked by H3K4me3 was unexpected given biochemical experiments documenting the failure of NuRD to productively interact with H3K4 methylated peptides [28], [29]. Importantly, the current data are in excellent agreement with CHD4 ChIP-seq experiments performed in thymocytes by Katia Georgopoulos and colleagues [4] and with ChIP-seq of exogenously expressed MBD3 in HeLa cells [30] and in murine ES cells [31]. MBD3 enrichment with active histone marks at regions with the characteristics of enhancers underscores the surprising association of NuRD with the active fraction of the genome, in agreement with reports on CHD4 localization in K562 cells [13] and in murine ES cells [10]. Given that the half-life of acetylated histones in the active fraction of the genome is less than 5 minutes [32], it is not surprising that histone deacetylases, including NuRD, may accumulate there [33]. Presumably, the dynamic equilibrium between the acetylated and deacetylated state for histones, or other chromatin associated factors, is an important determinant of promoter/enhancer function and its regulation.

While association of MBD3 with active promoters was abundant in our data, we also observed accumulation in regions with local chromatin marks diagnostic of transcriptional repression. A significant number of promoters marked by H3K27me3 in MCF-7 cells also bore a peak of MBD3, in agreement with ChIP-seq for CHD4 in murine ES cells [11]. Somewhat surprisingly, we did not observe substantial colocalization of MBD3 with H3K9me3 (although we did observe that a substantial proportion of promoters bound by H3K9me3 are also bound by MBD3), despite extensive biochemical data documenting specific interaction of the PHD finger domains of CHD4 with this mark [34]. While we do not completely understand the nature of this discrepancy, it may reflect the propensity of this histone mark to be localized in the repetitive fraction of the genome. The biochemical data also indicate high affinity interaction of CHD4's PHD fingers with H3K9 acetylation [35], which agrees very nicely with our ChIP-seq data. It is interesting to speculate that association of the PHD1/2 domain of CHD4 with different modifications, both of which change the physical properties of a single lysine residue on histone H3, may be instrumental in directing NuRD to regions of the genome with completely opposing functional states.

The methyl CpG binding domain family is intimately tied to cytosine modification [36]. MBD3 is a most interesting member of this family, being a bona fide methyl-CpG binding factor in some, but not all, taxa [37]. Whether MBD3 can sense cytosine modification remains a matter of some contention in the literature; some investigators describe interactions with 5-hydroxymethyl C [9], others do not [21], [22], [31]. Here, we describe enrichment for MBD3 at CpG islands that have extremely low levels of cytosine modification as measured by reduced representation bisulfite sequencing which does not distinguish between methylation and hydroxymethylation. We interpret this data as supporting the model that mammalian MBD3 does not recognize cytosine methylation or hydroxymethylation [21], [31] and is preferentially bound at CpG islands with low levels of cytosine modification.

Collectively, MBD3 localization supports roles for NuRD complex in regulation of chromatin structure and/or protein modification status at promoters of active genes, at enhancers, at stably repressed genes, and in bodies of actively transcribed genes. Functional data reported here document a role for MBD3, and by extension NuRD, in nucleosome organization, a critical determinant of chromatin structure. Further, they predict novel functions that remain to be described. These predictions clearly indicate that models describing NuRD as a static corepressor are inadequate in the face of emerging genomic data. Rather, it seems likely that NuRD is involved at multiple levels in modulation of epigenetic features to facilitate chromatin biology. Recently, whole-exome sequencing revealed high-frequency deletion of a short segment of chromosome 19 containing the *MBD3* locus in uterine serous carcinoma and frequent point mutations in CHD4 in serous endometrial tumors, suggesting fundamental functions of NuRD in primary tumors [38], [39]. Future challenges for the field include defining modes of local enrichment at specific genomic features as well as functional studies to describe the nature and extent of enzymatic and non-enzymatic actions of NuRD complex on the chromatin fiber.

## Methods

### Cell culture

MCF-7, MDA-MB-231, and 293T cells were obtained from the American Type Culture Collection and cultured at 37°C, 5% CO2 in Dulbecco's Modified Eagle Medium/Nutrient Mixture F-12 (DMEM/F-12) Media containing 10% fetal bovine serum supplemented with penicillin-streptomycin.

### Plasmids and virus construction

DamID lentiviral vectors, pLgw-RFC1-V5-EcoDam and pLgw-V5-EcoDam were kindly provided by Dr. Bas van Steensel, Netherlands Cancer Institute. Human MBD3 cDNA (BC043619) was amplified and cloned into pENTR/D-TOPO and then recombined by an LR-reaction into destination vector pLgw-RFC1-V5-EcoDam (pLgw-MBD3-V5-EcoDam). Retroviral knockdown constructs, pSMP-Luc (Addgene plasmid 36394) and pSMP-MBD3_3 (Addgene plasmid 36373) were obtained from Addgene. All constructs were verified by DNA sequencing. Lentivirus production and infection were performed as previously described [40], using pLgw-MBD3-V5-EcoDam (MBD3-Dam) or pLgw-V5-EcoDam (Dam-only). Retrovirus production and infection were performed as described [41], using pSMP-Luc or pSMP-MBD3_3.

### DNA adenine methyltransferase identification (DamID) and microarrays

The DamID experiments were carried out as previously described [42]. Briefly, MCF-7 or MDA −231 cells were seeded into 6-well plates. Seventy-two hrs after infection, genomic DNA was isolated using Qiagen DNeasy tissue kit. Genomic DNA (2.5 µg) was digested with Dpn I followed by adaptor ligation. The ligated product was digested with Dpn II and amplified by PCR. One microgram amplified product was labeled using Dual-color DNA Labeling kit (Nimblegen) according to manufacturer's protocol and then hybridized to Nimblegen 2.1M Deluxe promoter array or human 2.1 M Whole-Genome Tiling array (Array 5 of 10 covering chromosomes 6–8) and washed following the manufacturer's directions. The slides were scanned using a DNA microarray scanner (G2565BA; Agilent Technology) and the images were processed with the Nimblegen software.

### DamID microarray data normalization

A two-step normalization approach was used, where the first step is designed to correct for GC bias and dye bias within a chip (intrachip correction) and the second step corrects for variations across chips (interchip correction). The first step was within-chip normalization. First, all probes were binned according to their GC content. The GC content was computed as a ratio of C and G nucleotides to the total number of nucleotides in the probe sequence. The overall variability in GC content values was used to compute bin width according to zero-stage rule [43], [44]. These bin widths are proven to be approximate L2 optimal; i.e., they minimize mean integrated square error. The bins with fewer probes were then merged so that each bin contains at least 500 probes. Within each bin, Lowess regression was used to predict log-transformed cy5 values as a smooth function of log-transformed cy3 values [45], [46]. The scaled (median of absolute residuals is used for scaling) difference between observed and predicted log(cy5) values were used as normalized signal.

The second step was between-chip normalization. Once the data were corrected for dye and GC bias as described in the first step, we employed quantile normalization independently for each histone mark and DNA methylation to correct for between-chip variation.

### Identifying MBD3 binding peaks

The differentially bound “peak” regions for each cell type comparisons were identified using modified ACME algorithm that allows for spooling data across replicates [46]. This algorithm like ACME, depends on three user-specified tuning parameters: window size (*w*), signal threshold (*s*) and p-value threshold (*p*).

To identify MBD3 bound peaks, we first compute the number (*x*) of signal values within window of size *w* (centered at probe) that are greater than 100*s* ^th^ percentile across all replicates. Next, we compute enrichment p-value for probe using hypergeometric distribution as following
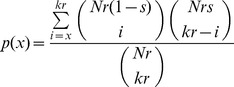
where *N* denotes total number of probes, *k* denotes number of probes in window and *r* denotes the number of replicates.

Finally, the peaks are identified as runs of enrichment p-values that are less than p-value threshold (*p*). The analysis presented here correspond to signal threshold (*s* = 0.95), window size (*w* = 2000) and p-value threshold (*p* = 0.001) with peaks containing less than six probes excluded.

### Quantitative functional analysis (qFA)

For each set of differentially bound and cell-specific gene signatures we performed Fisher's exact test to assess enrichment of gene-sets from Molecular Signature Database (MSigDB, version3.0, Broad Institute) and other published Cancer gene sets. The resulting significance p-values were subjected to Benjamin-Hochberg (FDR) multiple test correction.

### DamID and ChIP-seq peaks overlap analysis

We employed chromosome bound circular permutations test described in [46] to assess whether the observed overlap between two sets of genomic intervals (e.g. peaks) is significantly enriched or depleted compared to expected overlap. To perform overlap analysis, we used disjoined probes from the promoter-array as a unit of overlap. The ‘member status’ (ie. whether the disjoined probe belongs to a genomic region of interest or not) of disjoined promoter probes in one of genomic interval set was permuted 20,000 times using chromosome bound circular permutation as following. For each permutation a randomly generated number drawn from uniform distribution between 1 and number of disjoined probes from the analyzed chromosome was used to shift the membership status of all disjoined probes within a chromosome. The permutations resumed at the beginning of chromosome when the shift of status exceeded number of disjoined probes available in a chromosome. The odds-ratio of enrichment/depletion, corresponding significance p-value and 95% confidence interval were calculated by comparing observed overlaps and average overlap across all 20,000 permutations.

The above approach was utilized to assess enrichment/depletion of overlap in significant DamID peaks (modified ACME peaks with at least 6 promoter probes) and MBD3 ChIP-seq peaks (peaks with fold change> = 2 and false discovery rate< = 0.00001).

We also utilized the above approach to assess enrichment/depletion of overlap in significant cell specific DamID peaks (uncommon peaks in MCF-7 and MDA-231 cells) with promoter regions (3 kb upstream and 3 kb downstream of TSS) of Luminal vs. Basal breast cancer differentially expressed genes from Charafe et al [47].

### ChIP and ChIP-seq library preparation

ChIP experiments for MBD3 were performed as previously described with several modifications [17], [18]. MCF-7 or MDA-231 cells were crosslinked with 1.5 mM disuccinimidyl glutarate (Thermo Scientific) for 45 min at room temperature followed by 1% formaldehyde (Sigma) for 10 min at room temperature. Fixation was quenched by 125 mM glycine. Cells were washed twice with ice-cold PBS and stored at −80°C. Cells were thawed on ice and resuspended with 270 µL lysis buffer [50 mM Tris-HCl (pH 8.0), 10 mM EDTA, 1% SDS and protease inhibitor]. After incubation on ice for 5 min, cells were sonicated with Bioruptor for 6 cycles of 30 sec on and off at HIGH setting. Samples were centrifuged at 16.1 kg for 5 min at 4°C. Supernatant was collected into new tube and pellet was resuspended with 150 µL lysis buffer. The resuspended pellets were sonicated with Bioruptor for 4 cycles of 30 sec on and off at HIGH setting. Samples were centrifuged at 16.1 kg for 5 min at 4°C and supernatant were combined. 100 µL supernatant was diluted 10 times with IP buffer [16.7 mM Tris-HCl (pH 8.0), 1.2 mM EDTA, 0.01% SDS, 1.1% TritonX-100, 167 mM NaCl, 5 mg/mL BSA and protease inhibitor] and incubated with 4 µg of control rabbit IgG (Santa cruz) or anti-MBD3 antibody (Abcam; ab91458) at 4°C overnight. Samples were further incubated with 20 µL of Dynabeads Protein A and G at 4°C for 1.5 hr. The beads were washed once with IP buffer, twice with RIPA buffer [25 mM Tris-HCl (pH 7.6), 150 mM NaCl, 1% NP-40, 1% sodium deoxycholate, 0.1% SDS], twice with high salt RIPA buffer (1∶9 dilution of 5M NaCl with RIPA buffer), twice with LiCl buffer [20 mM Tris-HCl (pH 8.0), 250 mM LiCl, 1% NP-40, 1% sodium deoxycholate, 2 mM EDTA] and twice with TE buffer [10 mM Tris-HCl (pH 8.0), 0.1 mM EDTA]. Washed beads were resuspended with elution buffer (1% SDS and 0.1 M sodium bicarbonate) and incubated at 65°C for 45 min. Eluted DNA was adjusted to 300 mM NaCl and incubated with RNase at 65°C for 4 hr followed by incubation at 65°C for 1 hr with proteinase K. DNA was purified with a MinElute PCR purification kit (QIAGEN). Sequencing libraries were prepared with Truseq DNA kit or Nextera XT kit (following the manufacturer's protocols).

MBD3 ChIP-seq and Input libraries were sequenced using Illumina GAIIx technologies at the NIH Intramural Sequencing Center (MCF-7 MBD3 ChIP, MCF-7 Input and MDA-231 Input) or using MiSeq technologies at the NIEHS Epigenomics Core Facility (MDA-231 MBD3 ChIP and replicate Input). Standard Illumina CASAVA 1.8 utilities were used to generate .fastq output files. All libraries were sequenced as single end 36mers.

ChIP experiments for H3K4me3 in MDA-231 cells were carried out using a Magna ChIP kit (Millipore) following the manufacturer's suggested protocols. For each ChIP experiment, 5×10^6^ MDA −231 cells were crosslinked with 1% formaldehyde at 37°C for 10 min. The anti-H3K4me3 (Upstate, no. 04-745) antibody was used. The ChIP-seq libraries were prepared from 10 ng of both Input and ChIP DNA samples using a ChIP-seq sample preparation kit (Illumina) according to the manufacturer's protocol.

MDA-231 H3K4me3 ChIP-seq and Input libraries were sequenced using Illumina GAII technologies at the University of Missouri DNA Core Facility. For these libraries Illumina CASAVA 1.3 utilities were used to generate .fastq output files. All libraries were sequenced as single end 36mers.

### ChIP-seq analysis

Public MCF-7 data used for analyses included: H3K4me3 (GSM945269), H3K9me3 (GSM945857), H3K27ac (GSM945854), H3K27me3 (GSM970218), Reduced Representation Bisulfite (GSM683787), and triplicate expression data (GSM425734, GSM425735, GSM425736) and (GSM425737, GSM425738, GSM425739).

Sequenced reads from MDB3 ChIP-seq and Input libraries were combined for replicate samples and filtered based on a mean base quality score <20. Filtered reads were then aligned to the human reference genome (UCSC assembly hg19, GRCh37) using the Bowtie short-read alignment program (v0.12.8 employing parameters −v 2, −m 1) to retain reads mapped to unique genomic locations with at most 2 mismatches. Only non-duplicate reads were used in subsequent peak calling analyses and the generation of coverage tracks. To make the coverage tracks, aligned reads were extended at the 3′ end to a length of 300 bases (the expected genomic fragment size), and bigWig files were generated to visualize aggregate genomic coverageMBD3 peaks for each cell type were identified using SICER with a FDR threshold of 0.001 and the following parameters (redundancy threshold = 1, window size = 200, gap size = 600, fragment size = 300). Basic quality control of MBD3 ChIP-seq data (ours and others) is provided in Table S5.

### Annotation of MBD3-binding peaks

hg19 RefSeq transcript model definitions were downloaded from UCSC. Transcripts covering duplicated genomic loci [have exactly same transcription start site (TSS) and end site (TES)] were excluded yielding 31,723 RefSeq transcripts. ChIP-seq peaks were defined as associated with genomic features if they intersect at least 1 bp. Genomic features queried included exons, introns, TSS, TES, promoters (+/− 3 kb from TSS), gene bodies (TSS to TES), and intergenic regions (not in gene body).

### Binding profiles relative to gene features

From the set of 31,723 RefSeq transcripts, we selected full-length gene bodies (i.e. from the transcript start to the 3′ transcript end) larger than 2 kb.

Read counts were averaged in 70 bp windows for regions +/− 5 kb from TSS. Within the gene bodies, reads were averaged in windows equal to 1% of the gene length. All window read counts were normalized by the total number of bases in each window and the length of the window.

### Classification of promoters

The CpG contents and the ratio of observed versus expected CpG dinucleotides were determined in a 1,001 bp window around TSS (+/− 500 bp) as described [48]. Promoter categories were then classified into 3 categories as follows: HCPs (high-CpG promoters) contain a 500 bp area with CpG ratio above 0.75 and GC content above 55%; LCPs (low-CpG promoters) do not contain a 500 bp area with a CpG ratio above 0.48; and ICPs (intermediate CpG promoters) are neither HCPs nor LCPs.

### Heatmap

Heatmaps represent transcript expression, binding profiles of MBD3 and H3K4me3 in MCF-7 and MDA-231 cells at promoter regions (−7 to +3 kb relative to TSS) and are sorted by MBD3 binding profiles. For DamID data, we iteratively filtered genes as followed: 1) Downloaded hg18/refseq gene table from UCSC browser site (with date stamp October 20, 2009). 2) 31,451 genes from chr1-22 and chrX and chrY were retained. 3) 24,983 genes with non-missing gene expression signal were retained. 4) 15,292 genes with at least one microarray probe for each TSS bin were retained. 5) 11,872 genes with unique TSS were retained. 6) 8,207 genes with no 7 kb upstream and no 3 kb downstream neighbors were used in heatmap. For ChIP-seq data, we filtered genes as followed: 1) Transcripts with duplicated genome locus were eliminated. 2) Transcripts were selected with a MAD (Mean Absolute Deviation) higher than 1.0, to further eliminate transcripts with low variability around promoter regions in MBD3-binding profiles; 25,569 transcripts (MCF-7) and 23,605 transcripts (MDA-231) were retained and used in the heatmap.

### Signal for MBD3 vs histone modification marks

Raw sequencing datasets for MBD3 and histone modifications H3K4me3, H3K27me3, H3K27ac, and H3K9me3 from ENCODE were filtered, mapped, deduplicated, and extended (to 300 bp) as described above. RPKM thresholds for establishing presence or absence of each mark were determined following the method described by Whyte et al for detecting super-enhancers [49], but evaluating over all non-overlapping 1 kb genomic bins rather than called peaks. If one or more 1 kb bin (evaluated with step size 100) in the region +/− 3 kb of an annotated RefSeq TSS had RPKM above the established threshold, presence of MBD3 or histone mark was assigned for that TSS. Comparison of MBD3 signal with H3K4me3, H3K27ac, H3K9me3, or H3K27me3 at all TSS is shown by Venn diagram.

### Reads distribution around peak center

Called MBD3-binding peaks in MCF-7 cells were separated according to whether they overlap with a RefSeq promoter region (TSS +/− 3 kb). Then a region with 3 kb flanking the center of peaks was extracted accordingly. Reads from MBD3, H3K4me3 and H3K27ac profiles were averaged in 60 bp windows for the above region and normalized by the total mapped reads.

### Methylation status of CpG islands associated with MBD3-binding peaks

CpG islands annotation was downloaded from UCSC hg19 genome, and CpG islands were separated according to whether they overlap at least 1 bp with the MBD3-binding peaks. Reduced Representation Bisulfite sequencing data in MCF-7 cells (GSM683787) was downloaded and the methylation levels of the CpG islands associated with MBD3-binding peaks were calculated as the follows: percentage of methylation = total sequenced reads being methylated/total reads being sequenced.

### Analysis of ChIA-PET data

The interaction dataset, part of the Pol II Chromatin Interaction Analysis with Paired-End Tag data (Pol II ChIA-PET), GSM970209, were downloaded from GEO database. Then the data were filtered to keep the PET pair ends that have at least one end overlapped with the TSS loci (+/− 3 kb). Among the retained PET pair ends, each end in the PET pair ends was examined as follows: 1) Assign the nearest hg19 Refseq gene if this end is overlapped with TSS loci (+/− 3 kb); 2) Assess if this end was overlapped with the MBD3 peaks; 3) Count the reads from MBD3 ChIP-seq data within the region that is 500 bp flanking the center of this end. The reads were normalized to the total mapped reads.

The data are displayed as follows: 1) Sort the read number at TSS end of the filtered ChIA-PET pair ends in a descending order; 2) Plot the sorted read number in upper panel: y-axis is the sorted read number and x-axis is the sorted PET pairs; 3) Plot the read number in lower panel: y-axis is the corresponding read number in the other end and x-axis is the sorted PET ends in the same order as in the upper panel.

### Chromatin conformation capture (3C)

3C experiment was performed as previously described [25]. Briefly, MCF-7 cells were crosslinked by 1% formaldehyde for 10 min at room temperature and fixation was quenched by 125 mM glycine. Cells were resuspended with lysis buffer (10 mM Tris-HCl, 10 mM NaCl, 0.2% NP40) and incubated on ice for 15 min. The pellet was resuspended with 1.2×NEB2 buffer and incubated at 65°C for 15 min and followed by digestion with EcoRI overnight. Digested chromatin was ligated with T4 DNA ligase (NEB) at 16°C for 2 hr. Purified 3C template (200 ng) was amplified with iQ SYBR mix (BioRad).

### Genome-wide nucleosome mapping using micrococcal nuclease digestion followed by massively parallel sequencing (MNase-seq)

Mononucleosomal DNA were prepared from MCF-7 cells (Luc or MBD3 knockdown) as previously described with some modifications [50]. Briefly, nuclei were collected from 5×10^5^ cells and digested by MNase (1.25, 2.5, or 5 units) for 5 min at 37 °C. Mononucleosomal DNA were collected by gel size selection and pooled. Sequencing libraries were prepared from 1 µg of pooled DNA with TruSeq DNA Sample Preparation Kit. The resulting libraries were sequenced on a HiSeq 2000 (Illumina) as paired end 101mers. To ensure that low quality reads were excluded from the analysis, the raw sequence reads were filtered to remove any entries for which either read in a pair had mean base quality score <20. Filtered reads were aligned to the human genome (GRCh37/hg19) via Bowtie (v0.12.8 with parameters -m 1 –best –strata –chunkmbs 1024 -I 0 -X 1000) [51]. Multiple sequencing lanes from the same MNase library were merged prior to downstream analysis, and duplicate reads per library were removed using MarkDuplicates.jar from the Picard tools package (v1.86) (http://picard.sourceforge.net). To capture only sequenced reads corresponding to the size of a mononucleosome, filtering was applied to retain only fragments 120–180 bases in length; 90–95% of mapped, deduplicated read pairs per library passed this filter. Each mapped read pair was then converted to a single point at the midpoint of the fragment. The two replicate libraries per condition were merged, and ‘bedGraph’ files were generated (via BEDtools, version 2.17.0 [52]) for visualization of genomic distribution of mapped fragments.

Metagene plots of nucleosome occupancy were generated with mapped MNase-seq fragments downsampled to the same read count per condition (N = 156,822,505). Counts per position were aligned relative to each annotated RefSeq TSS, with the RefSeq loci split into quartiles based on the number of MBD3 ChIP-seq reads mapped to the region +/− 3 kb of the given TSS. The positional counts in each dataset (shMBD3 or shLuc per quartile) were normalized by the mean score over the region +/− 5 kb of TSS, then smoothed with a moving average (period = 50).

### Data access arrangements

The microarray data and sequence data from this study are available at the NCBI Gene Expression Omnibus (GEO) under accession number, GSE44737, GSE 44776, and GSE51097.

## Supporting Information

Figure S1 Validation of MBD3-Dam construct. A. pLgw-MBD3-V5-EcoDam was transfected to HeLa cells and MBD3-Dam protein was detected by anti-V5 tag antibody. B. pLgw-MBD3-V5-EcoDam was transfected to HeLa cells and the localization of MBD3-Dam protein was determined by immunofluorescence using anti-V5 tag antibody. Nuclear was stained with DAPI.
(TIF)

Figure S2 Comparison of DamID and ChIP-seq data. Representative results for CDH1 and PLS3 loci are shown.
(TIF)

Figure S3 Genome browser view of exemplar CpG island promoters that overlap a peak of MBD3. CpG islands are depicted below the tracks. Methylation or hydroxymethylation data (RRBS, Myers/HAIB, GEO accession number GSM683787, ENCODE Project Consortium 2011) is displayed below the tracks. Color coding of methylation data is indicated in the figure.
(TIF)

Figure S4 MBD3 peaks in intergenic regions are in physical proximity to promoter. Physical proximity of promoter region and distal MBD3 bound sites are detected by Chromosome Conformation Capture (3C) in MCF-7 cells. The promoter region of NRIP1, NR2F2, and MASTL were examined.
(TIF)

Figure S5 Validation of MBD3 knockdown in MCF-7 cells. Western blot shows MBD3 expression levels in control (shLuc) and MBD3 knockdowned cells. Histone H3 is used as a loading control.
(TIF)

Figure S6 MBD3 impacts nucleosome density at bound promoters and gene bodies. TSS's were binned into 4 equal sized bins by MBD3 occupancy level. Nucleosome position and density were plotted as described in Methods using data derived from control (shLUC) and MBD3 depleted (shMBD3) cells.
(TIF)

Table S1 Gene set enrichment in cell-type specific MBD3 DamID peaks.
(DOC)

Table S2 DamID vs ChIP-seq peaks comparison. Peak overlap between DamID and ChIP-seq was calculated with chromosome bound circular permutation method (see Methods).
(DOC)

Table S3 Gene set enrichment in cell-type specific MBD3 ChIP-seq peaks.
(DOC)

Table S4 Quality control of MNase-seq data.
(XLSX)

Table S5 Quality control of MBD3 ChIP-seq data. Ours and recently published MBD3 ChIP-seq data (GSM1006708, GSM786608, GSM786609, GSM972974, and GSM972974) were mapped with Bowtie (v0.12.8 employing parameter; -m 1) to identify reads mapped to unique genomic locations.
(XLSX)
