# Supplementary figures and images for: MBD3 Localizes at Promoters, Gene Bodies and Enhancers of Active Genes
Source: PLoS Genet. 2013 Dec 26;9(12):e1004028. doi: 10.1371/journal.pgen.1004028 (PMC3873231; doi:10.1371/journal.pgen.1004028)

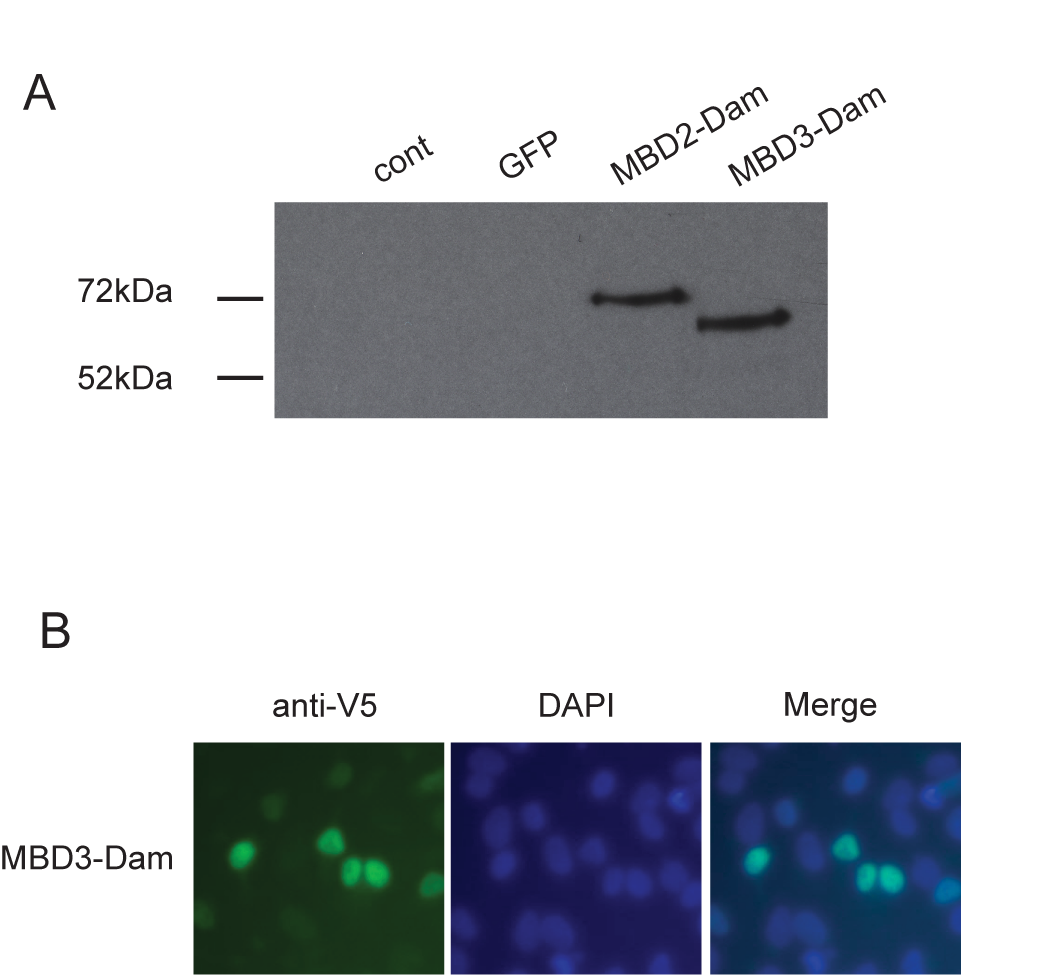

Supplement: Figure S1 — Validation of MBD3-Dam construct. A. pLgw-MBD3-V5-EcoDam was transfected to HeLa cells and MBD3-Dam protein was detected by anti-V5 tag antibody. B. pLgw-MBD3-V5-EcoDam was transfected to HeLa cells and the localization of MBD3-Dam protein was determined by immunofluorescence using anti-V5 tag antibody. Nuclear was stained with DAPI. (TIF) [file pgen.1004028.s001.tif]

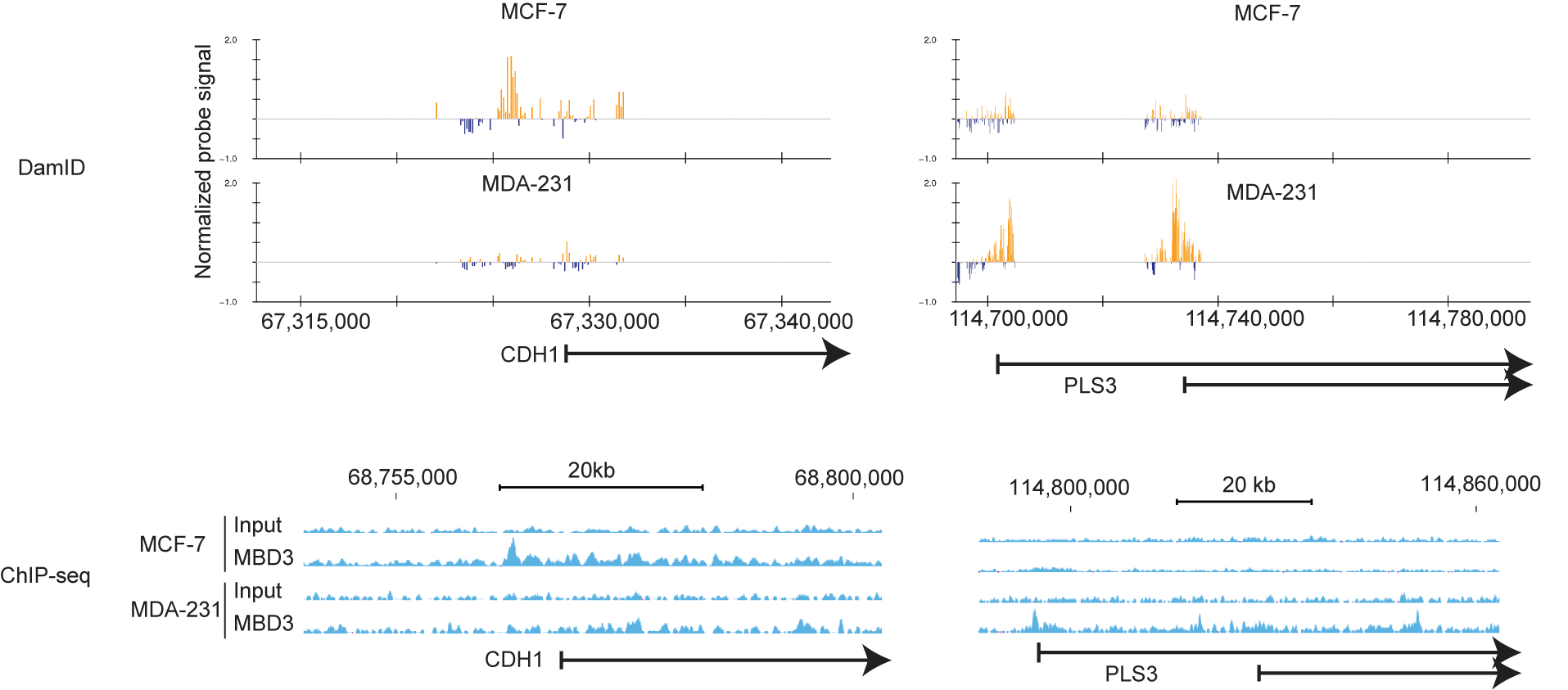

Supplement: Figure S2 — Comparison of DamID and ChIP-seq data. Representative results for CDH1 and PLS3 loci are shown. (TIF) [file pgen.1004028.s002.tif]

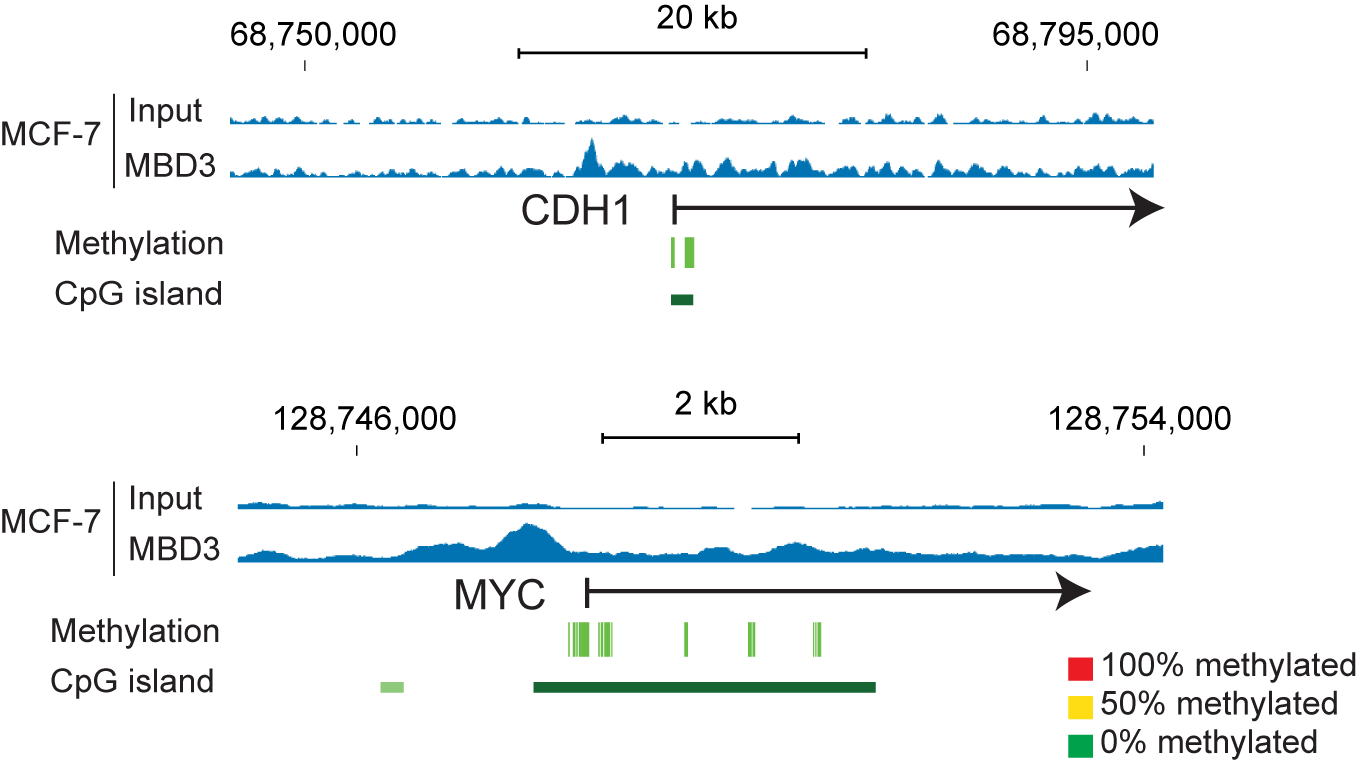

Supplement: Figure S3 — Genome browser view of exemplar CpG island promoters that overlap a peak of MBD3. CpG islands are depicted below the tracks. Methylation or hydroxymethylation data (RRBS, Myers/HAIB, GEO accession number GSM683787, ENCODE Project Consortium 2011) is displayed below the tracks. Color coding of methylation data is indicated in the figure. (TIF) [file pgen.1004028.s003.tif]

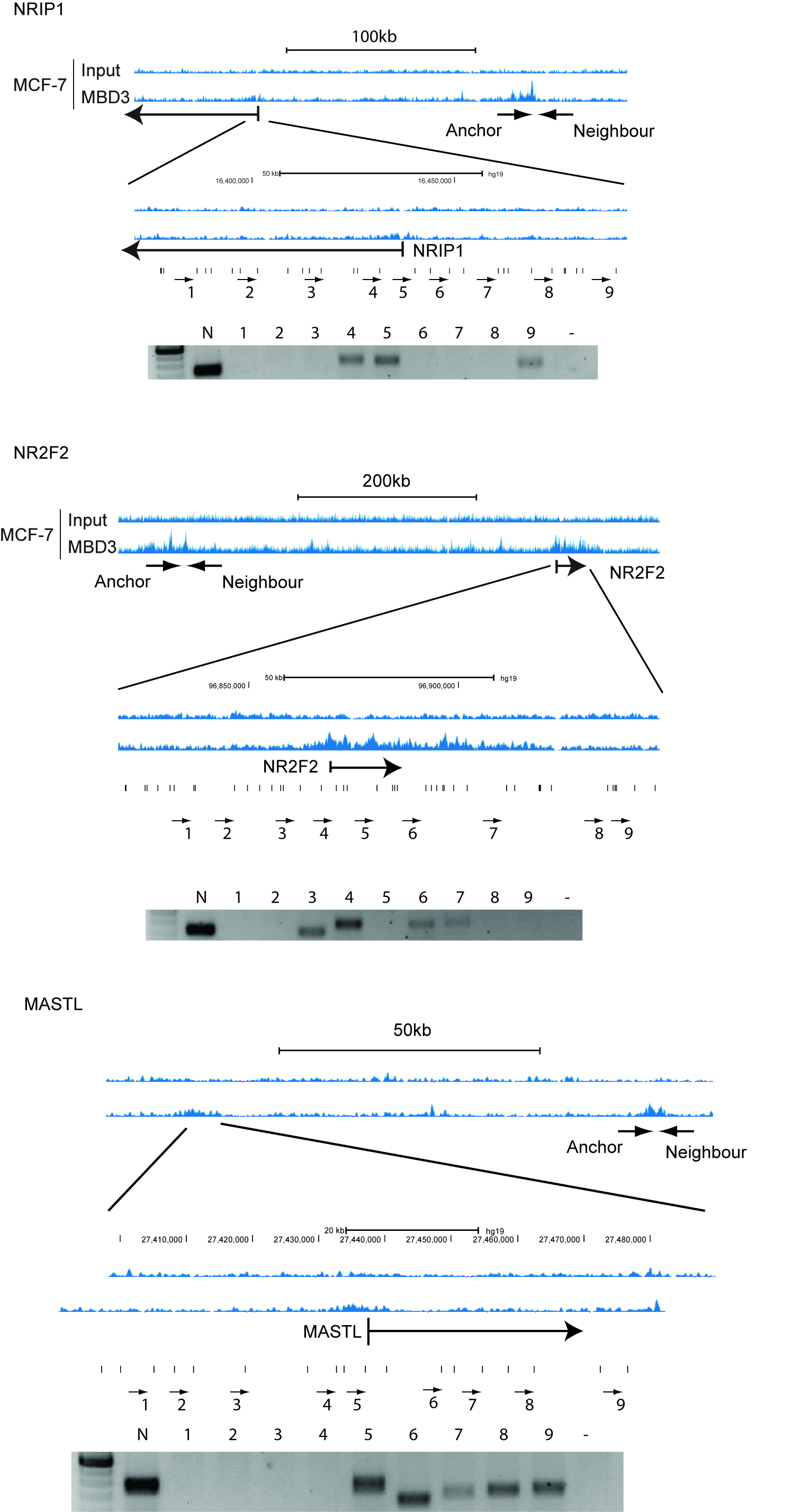

Supplement: Figure S4 — MBD3 peaks in intergenic regions are in physical proximity to promoter. Physical proximity of promoter region and distal MBD3 bound sites are detected by Chromosome Conformation Capture (3C) in MCF-7 cells. The promoter region of NRIP1, NR2F2, and MASTL were examined. (TIF) [file pgen.1004028.s004.tif]

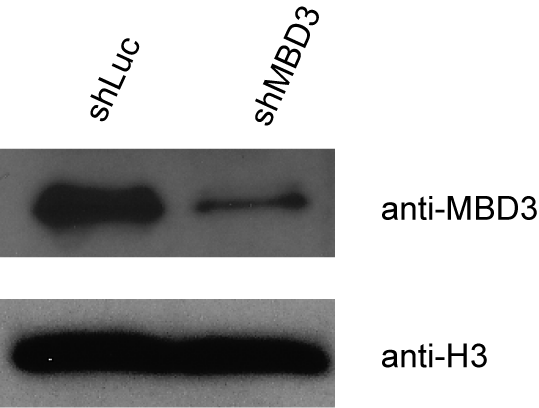

Supplement: Figure S5 — Validation of MBD3 knockdown in MCF-7 cells. Western blot shows MBD3 expression levels in control (shLuc) and MBD3 knockdowned cells. Histone H3 is used as a loading control. (TIF) [file pgen.1004028.s005.tif]

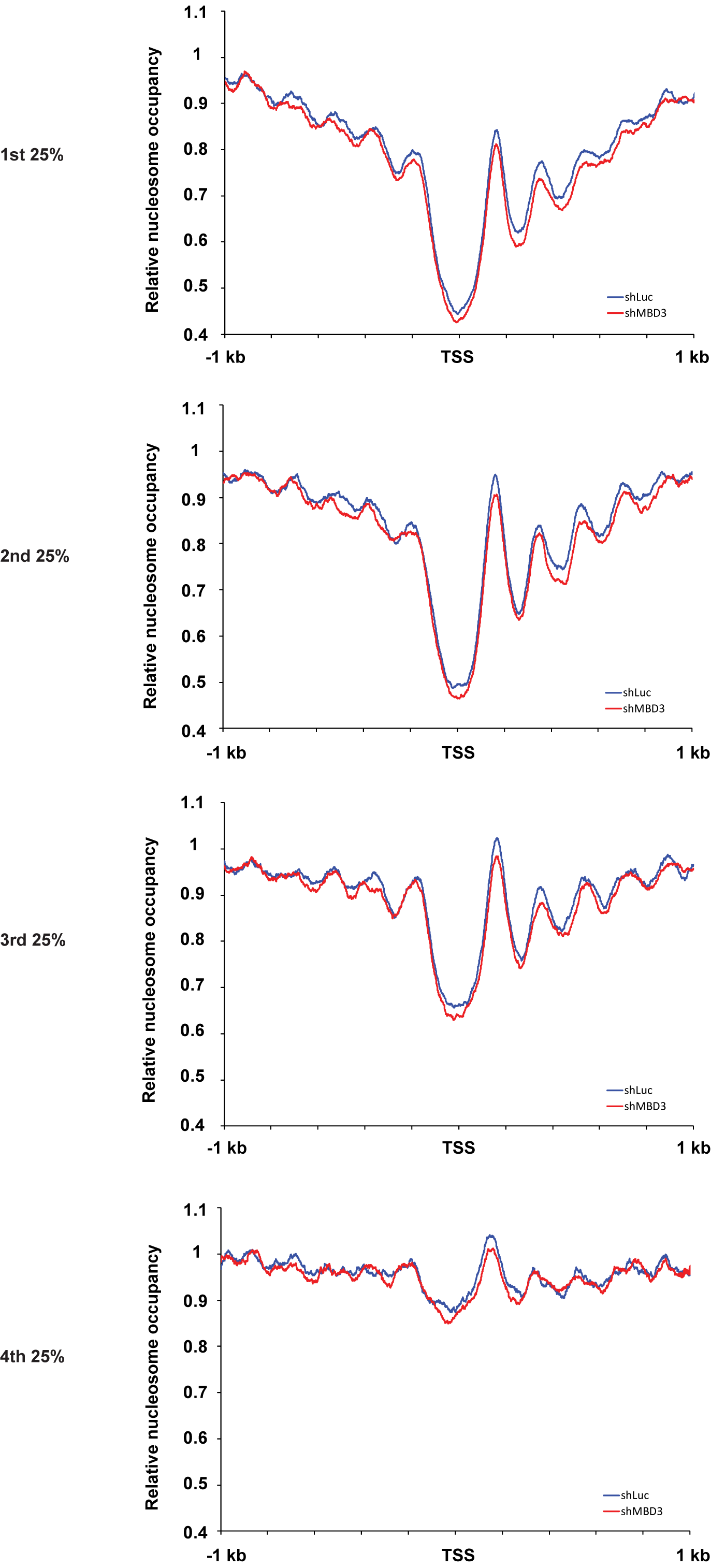

Supplement: Figure S6 — MBD3 impacts nucleosome density at bound promoters and gene bodies. TSS's were binned into 4 equal sized bins by MBD3 occupancy level. Nucleosome position and density were plotted as described in Methods using data derived from control (shLUC) and MBD3 depleted (shMBD3) cells. (TIF) [file pgen.1004028.s006.tif]
