# Supplementary material for: MBD3 Localizes at Promoters, Gene Bodies and Enhancers of Active Genes
Source: PLoS Genet. 2013 Dec 26;9(12):e1004028. doi: 10.1371/journal.pgen.1004028 (PMC3873231; doi:10.1371/journal.pgen.1004028)
Supplement: Table S1 — Gene set enrichment in cell-type specific MBD3 DamID peaks. (DOC) [file pgen.1004028.s007.doc]

| **Type** | **Gene set** | **Observed**  **Overlap**  **(%)** | **Expected**  **Overlap**  **(%)** | **Odds**  **Ratio** | **P-value** | **95% Confidence Interval** | |
| --- | --- | --- | --- | --- | --- | --- | --- |
|  |  |  |  |  |  | **Lower**  **Limit** | **Upper**  **Limit** |
| MCF-7 specific peaks | Luminal | 9.665 | 3.813 | 2.699 | < 5.000E-05 | 2.040 | 3.797 |
| MCF-7 specific peaks | Basal | 4.045 | 3.585 | 1.134 | 3.45E-01 | 0.881 | 1.532 |
| MDA-231 specific peaks | Luminal | 5.355 | 5.837 | 0.913 | 5.12E-01 | 0.718 | 1.208 |
| MDA-231 specific peaks | Basal | 16.108 | 6.015 | 3.000 | < 5.000E-05 | 2.417 | 3.822 |
