# Supplementary material for: MBD3 Localizes at Promoters, Gene Bodies and Enhancers of Active Genes
Source: PLoS Genet. 2013 Dec 26;9(12):e1004028. doi: 10.1371/journal.pgen.1004028 (PMC3873231; doi:10.1371/journal.pgen.1004028)
Supplement: Table S2 — DamID vs ChIP-seq peaks comparison. Peak overlap between DamID and ChIP-seq was calculated with chromosome bound circular permutation method (see Methods). (DOC) [file pgen.1004028.s008.doc]

| **Cell Type** | **Observed Overlap**  **(%)** | **Expected**  **Overlap**  **(%)** | **Odds**  **Ratio** | **P-value** | **95% Confidence Interval** | |
| --- | --- | --- | --- | --- | --- | --- |
|  |  |  |  |  | **Lower**  **Limit** | **Upper**  **Limit** |
| MCF-7 | 44.513 | 19.165 | 3.384 | < 5.000E-05 | 3.149 | 3.627 |
| MDA-231 | 36.193 | 11.592 | 4.326 | < 5.000E-05 | 4.027 | 4.645 |
