# Supplementary material for: MBD3 Localizes at Promoters, Gene Bodies and Enhancers of Active Genes
Source: PLoS Genet. 2013 Dec 26;9(12):e1004028. doi: 10.1371/journal.pgen.1004028 (PMC3873231; doi:10.1371/journal.pgen.1004028)
Supplement: Table S3 — Gene set enrichment in cell-type specific MBD3 ChIP-seq peaks. (DOC) [file pgen.1004028.s009.doc]

| **Type** | **Gene set** | **Observed**  **Overlap**  **(%)** | **Expected**  **Overlap**  **(%)** | **Odds**  **Ratio** | **P-value** | **95% Confidence Interval** | |
| --- | --- | --- | --- | --- | --- | --- | --- |
|  |  |  |  |  |  | **Lower**  **Limit** | **Upper**  **Limit** |
| MCF-7 specific peaks | Luminal | 25.568 | 11.087 | 2.755 | < 5.000E-05 | 2.166 | 3.651 |
| MCF-7 specific peaks | Basal | 14.784 | 10.097 | 1.545 | < 5.000E-05 | 1.240 | 1.978 |
| MDA-231 specific peaks | Luminal | 2.082 | 2.065 | 1.008 | 9.72E-01 | 0.660 | 1.829 |
| MDA-231 specific peaks | Basal | 7.440 | 2.165 | 3.632 | < 5.000E-05 | 2.493 | 6.010 |
